# Fracture-décollement de l’épiphyse médiale de la clavicule: à propos de 6 cas et revue de la littérature

**DOI:** 10.11604/pamj.2016.25.19.8787

**Published:** 2016-09-22

**Authors:** Amadou Ndiassé Kassé, Sid’Ahmed Ould Mohamed Limam, Souleymane Diao, Jean Claude Sané, Babacar Thiam, Mouhamadou Habib Sy

**Affiliations:** 1 Service d’Orthopédie-Traumatologie de l’Hôpital Général de Grand Yoff, Dakar, Sénégal

**Keywords:** Luxation sterno-claviculaire, décollement épiphysaire, épiphyse claviculaire médiale, Sternoclavicular dislocation, epiphyseal separation, medial clavicular epiphysis

## Abstract

Le but de ce travail est de décrire les caractéristiques épidémiologiques et les différentes entités anatomo-cliniques de la fracture-séparation de l’épiphyse claviculaire médiale mais également de rapporter les résultats morphologiques et fonctionnelsde de la réduction sanglante suivie de l’ostéo-suture au fil non résorbable. Cinq garçons et 1 fille âgés en moyenne de 14 ans ont présenté un traumatisme fermé et isolé de la ceinture scapulaire. L’examen clinique et l’imagerie médicale surtout la TDM ont permis de poser le diagnostic de décollement épiphysaire, de classer le degré d’ossification de l’épiphyse claviculaire médiale en précisant le sens du déplacement, ainsi que la nature du décollement selon Salter-Harris. Une réduction sanglante suivie d’une ostéo-suture au fil non résorbable décimale n°1 a été réalisées chez 3 patients. Un patient a bénéficié d’un embrochage croisé. Les deux plus jeunes ont été traités orthopédiquement. Le déplacement du moignon claviculaire était antérieur chez 3 patients et rétro-sternal chez les 3 autres. Les formes postérieures ont été compliquées d’une odynophagie (n=2) et d’une compression asymptomatique de la veine sous Clavière (n=1). L’une des formes postérieuresétait associée a une fracture ipsilatérale du 1 / 3 médial de la clavicule. La consolidation a été obtenue chez tous les malades avec une mobilité de l’épaule conservée. La fracture-décollement de l’extrémité médiale de la clavicule mime au plan clinico-radiologique la luxation sterno-claviculaire. Elle peut être grave en raison du risque de compression viscérale et vasculaire dans sa forme postérieure. La tomodensitométrie reste irremplaçable pour un diagnostic précis. Notre préférence va à la réduction sanglante suivie d’une ostéo-suture au fil non métallique.

## Abstract (en)

This study aims to describe the epidemiological characteristics and the different anatomo-clinical entities of the fracture-separation of the medial clavicular epiphysis but also to relate the morphological and functional results of bloody reduction followed by osteosuture using non absorbable thread. Five boys and one girl (mean age 14 years) showed a closed and isolated shoulder girdle trauma. Clinical examination and medical imaging, especially CT scan, allowed the diagnosis of epiphyseal separation and to classify the degree of medial clavicular epiphysiseal ossification indicating the direction of displacement as well as the nature of displacement according to the Salter-Harris classification. Bloody reduction followed by osteosuture using non absorbable thread (No. 1 decimal) was performed in 3 patients. One patient underwent cross-pinning the two younger patients were treated orthopedically. The displacement of the clavicle stump was anterior in 3 patients and retro-sternal in 3 patients. Posterior forms were complicated by odynophagia (n = 2) and asymptomatic compression of the subclavian vein (n = 1). One of the posterior forms was associated with an ipsilateral fracture of the medial one third of the clavicle. Consolidation was achieved in all patients with preservation of shoulder mobility. The fracture-separation of the medial end of the clavicle mimes clinically and radiologically the sternoclavicular dislocation. It can be serious because of the risk of visceral and vascular compression in its posterior form. Tomdensitometry is irreplaceable for an accurate diagnosis. Our preference is for bloody reduction followed by osteosuture using non metallic thread.

## Introduction

Les lésions traumatiques de l’articulation sterno-claviculaire restent rares [1, 2]. Elles représentent 1 à 3% des traumatismes de la ceinture scapulaire [1]. Elles regroupent les entorses, les luxations sterno-claviculaires, la fracture du 1/3 médial et la fracture-décollement de l’épiphyse médiale de la clavicule. Cette dernière entité est souvent confondue avec la luxation sterno-claviculaire d’où son appellation de « fausse luxation » [3]. Ce travail vise à décrire les caractéristiques épidémiologiques et les différentes entités anatomo-cliniques de la fracture-séparation de l’épiphyse claviculaire médiale mais également à rapporter les résultats morphologiques et fonctionnels de son traitement en insistant sur l’intérêt de la suture ostéo-périostée au fil non résorbable.

## Méthodes

Cinq (5) garçons et 1 fille, âgés en moyenne de 14 ans (extrêmes 0 et 19ans), ont présenté au décours d’un accident ludique (4 fois), d’un accident de circulation routière (1 fois) et d’un obstétrical (1 fois) un traumatisme fermé et isolé de la ceinture scapulaire. L’examen clinique et l’imagerie médicale surtout la TDM ont permis de poser le diagnostic, de classer le degré d’ossification de l’épiphyse claviculaire médiale selon SCHMELING modifiée par KELLINGHAUS [4, 5], de préciser le sens du déplacement, ainsi que la nature du décollement selon SALTER-HARRIS. Outre ces formes anatomo-cliniques, d’autres ont été identifiées et classées suivant l’âge, la survenue d’une complication concomitante ou post-opératoire, l’existence d’une lésion associée. Le traitement a été orthopédique avecune contention par un bandage élastique et une écharpe soulageant l’épaule chez les 2 plus jeunes patients. Les 4 adolescents ont bénéficié d’une réduction sanglante suivie d’une ostéosynthèse par une suture ostéo-fibro-périostée (3 fois) et d’un brochage en « X » pour le dernier cas (Tableau 1).

**Tableau 1:** Nos patients (n=6)

Cas		1	2	3	4	5	6
Sexe		M	M	M	M	M	F
Age (ans)		17	19	4	16	17	0
Profession		Elève	Tailleur	Sans	Elève	Elève	sans
Coté atteint		D	G	G	G	D	D
							
Type d’accident		AcVC (Chute/ Lutte)	Violence Rixe)	AcVC (chute d’escaliers)	AC (véhicule & arbre)	AcVC (Chute/Lutte)	Traumatisme Obstétrical
DESC		H12	H2	H6	H3	H18	J2
Clinique		Douleur+tuméfaction	Douleur Odynophagie	Douleur Œdème local	Douleur+ tuméfaction	Douleur+ discrète tuméfaction	Douleur et déformation post natales
TDM	Déplacement	Antérieur et inferieur	Postérieur	Antérieur et supérieur	Postérieur	Postérieur	Antérieur et supérieur
	Schmelling modifiée par Kellinghauss	II C	II B	I	IIA	I	I
	Salter et Harris	I	I avec Fracture 1/3 externe clavicule	I	II	I	I
Complications			Compres sion vasculaire		Odynophagie		
Délai de PEC (jours)		4	3	3	3	6	2
Traitement		Ostéo suture	Ablation du 1 / 3 interne de la clavicule+ ostéosuture	Orthopédique	Ostéosuture	Brochage croisé	Orthopédique
Recul (mois)		14	14	14	14	7	18
Score d’ESKOLA		11	10	11	10	9	12

AcVC: Accident de la vie courante ; AC: Accident de la circulation; PEC: Prise en charge

### Technique chirurgicale

L’intervention a été réalisée sous anesthésie générale avec une intubation oro-trachéale. Le patient est installé en décubitus dorsal, un billot sous l’épaule ipsi-latérale. L’abord cutané est en « L renversé » centré sur l’articulation sterno-claviculaire. Les découvertes opératoires sont marquées par un décollement de l’épiphyse souvent pur (3/4 opérés), un dégantement périosté avec une position rétro-sternale du moignon claviculaire. La réduction a été obtenue dans ce déplacement postérieur de façon douce et progressive par une traction en dehors et en arrière de la clavicule saisie par une pince de Jayle. L’ostéosynthèse est réalisée par 2 à 3 points disposés en « X » avec une ligature non métallique et non résorbable en Polyester tressé de décimale 4. Pour la fracture associée, une résection du moignon claviculaire libre a été effectuée devant la difficulté de repositionnement du fragment. Pour ce dernier, le ligament costo-claviculaire a été préservé et une « tubulisation du fourreau périosté » a été effectué permettant en plus la stabilisation du bout libre de la clavicule. En post-opératoire, l’épaule est immobilisée dans un bandage type « Mayo » pour 4 à 5 semaines avec une auto-rééducation. Un chirurgien vasculaire était averti à chaque fois qu’un patient qui présente un déplacement postérieur était installé sur la table opératoire. Le score d’ESKOLA [6] a été utilisé pour l’évaluation clinique au dernier recul.

## Résultats

### Epidémiologie

La séparation de l’épiphyse médiale de la clavicule a été notée chez un nouveau né de 2 jours, un enfant de 4 ans et 4 adolescents âgés respectivement de 16, 17 et 19 ans avec une nette prédominance masculine 4 garçons pour une seule fille. Le coté droit a été atteint autant que le gauche. La chute avec réception sur le moignon de l’épaule était le mécanisme lésionnel constamment retrouvé à l’exception du seul cas obstétrical iatrogène. Les autres circonstances traumatiques étaient dominées par les accidents ludiques et 1 cas de violence inter-personnelle.

### Formes cliniques

**Figure 1:**
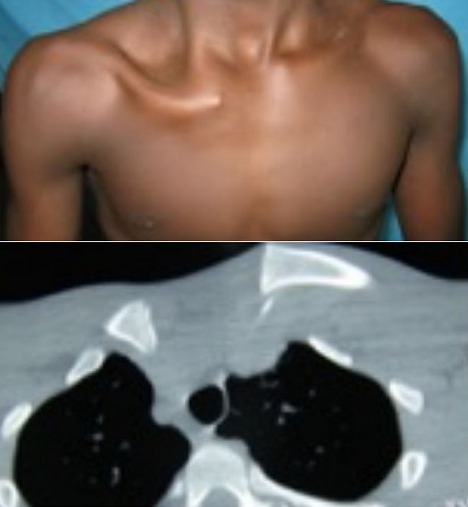
Fracture- décollement antérieure : Aspect clinique avec une saillie antéro-inférieure du moignon claviculaire et aspect tomodensitométrique avec un décollement type 1 de Salter et 2C de Schmelling

**Figure 2:**
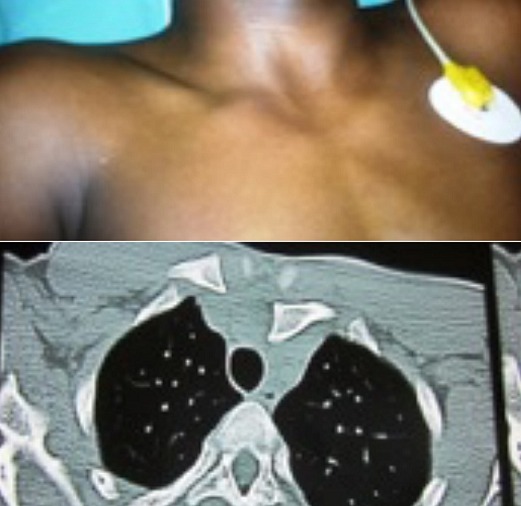
Fracture- décollement postérieure: aspect clinique avec une dépression rétro-sternale et aspect tomodensitométrique avec un décollement type 1 de Salter et 1 de Schmelling

**Formes anatomo-radiographiques:** l’ossification de l’épiphyse médiale de la clavicule a été classée selon **Schmelling** modifiée par **Kellinghauss** et était de type 1 chez 3 patients et de type 2 chez les 3 autres avec les sous-type A,B et C, une fois pour chacun. La classification du décollement épiphysaire selon **Salter-Harris** retrouvait une forme pure soit le type I dans 5 cas et le type II dans un cas. Le déplacement du moignon claviculaire était antérieur (Figure 1) chez 3 patients dont une fois vers le bas et rétro-sternal chez les 3 autres (Figure 2).

**Figure 3:**
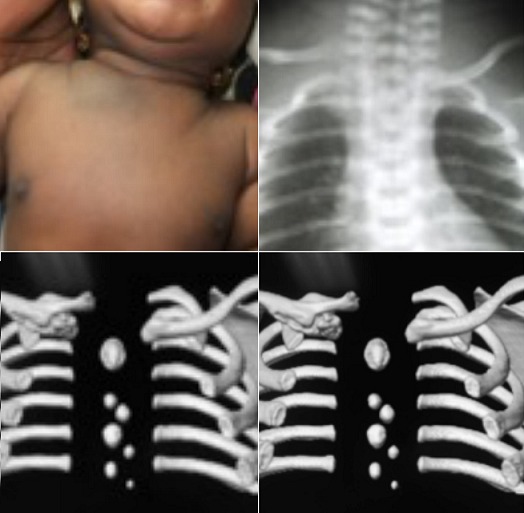
Forme néo-natale post traumatisme obstétrical: aspect clinique avec une saillie antéro-supérieure + Ecchymose base droite du cou; aspect radiographique avec une surélevation de la clavicule droite et scannographique avec un calluspériosté étendu et éxubérant mimant celui d’une fracture médio-diaphysaire de la clavicule

**Formes suivant l’âge:** un nouveau-né de 02 jours de sexe féminin nous a été présenté pour une fracture de la clavicule suite à un accouchement dystocique. L’examen clinique et la TDM ont permis de confirmer le diagnostic de cette forme néonatale (Figure 3). Les autres formes liées à l’âge ne présentaient pas de particularité propre.

**Formes compliquées:** les formes postérieures ont été compliquées d’une odynophagie (cas 4) et d’une compression asymptomatique de la veine sous-clavière par le 1/3 externe de la clavicule retourné sur lui-même (Cas 2). Cette compression a disparue avec l’abord du foyer et la levée de l’obstacle osseux.

**Figure 4:**
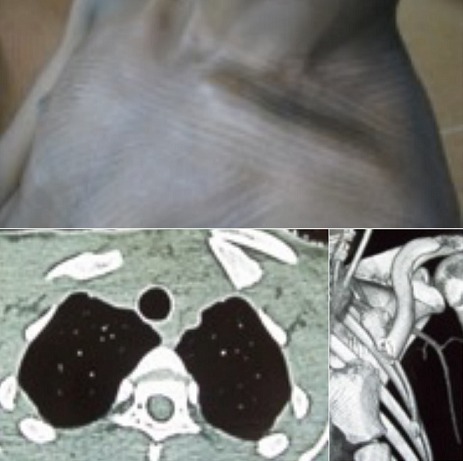
Forme associée à une fracture ipsi-latérale de la clavicule gauche: aspect clinique avec une saillie antérieure et dédoublement postérieur. TDM: fracture avec retournement clavicule sur elle-même et effet de masse sur le dome pleural. Aspect en 3D

**Formes associées:** l’une des formes postérieures était concomitante d’une entorse de l’acromio-claviculaire type I de Rockwood réalisant ainsi une clavicule flottante et l’autre d’une fracture ipsilatérale du 1 / 3 médial dela clavicule (cas 2) resté libre avec une rotation de près de 180°. Ce fragment était responsable de la compression de la veine sous-clavière gauche (Figure 4).

### Résultats thérapeutiques

**Figure 5:**
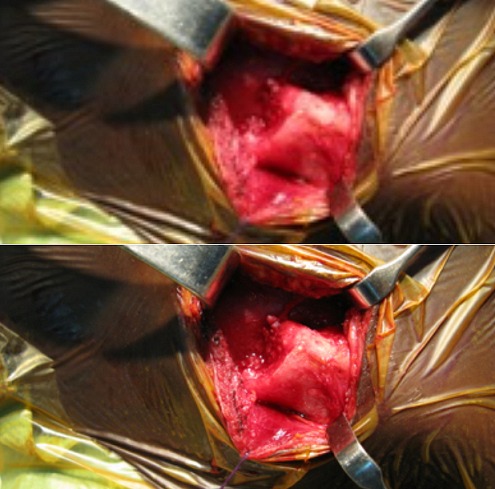
Les différentes étapes de la suture ostéo-périostée: découverte à l’ouverture du moignon claviculaire immédiatement sous la peau (forme antérieure). Découverte à l’ouverture du moignon claviculaire en situation rétro-sternale (forme postérieure)

**Figure 6:**
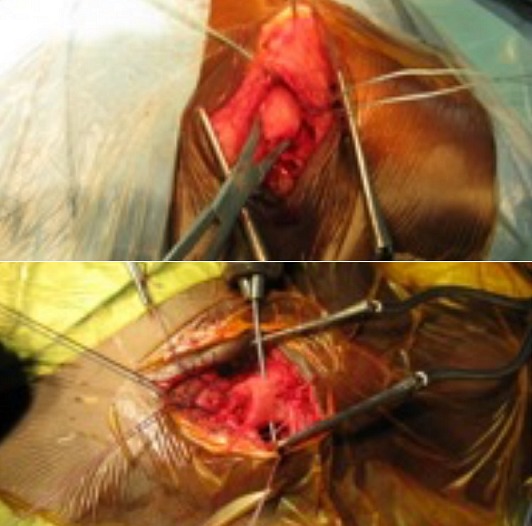
Les différentes étapes de la suture ostéo-périostée: réduction à l’aide de la Pince de Jayle. Perforation osseuse à l’aide du mandrin de Jacobs

**Figure 7:**
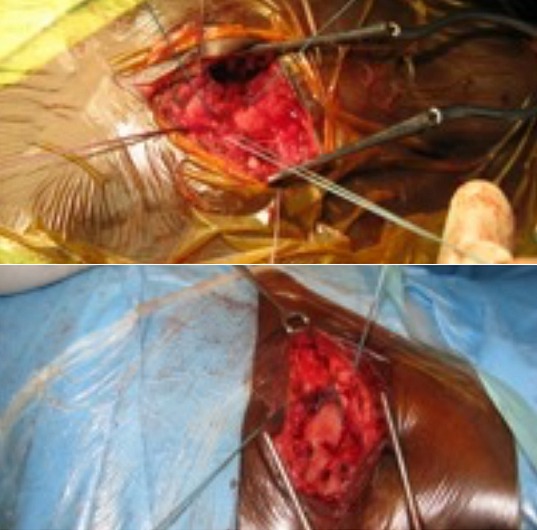
Les différentes étapes de la suture ostéo-périostée. Sutures osseuse et périostée: passage des fils. Sutures en place par du fil non métallique tressé déc 4

Chez le nouveau-né et le garçon de 4 ans, le traitement a été orthopédique d’autant que le déplacement était antérieur et qu’ils ont été vus tardivement. La consolidation a été obtenue avec un remodelage complet voire même un modelage au scanner pour le nouveau-né alors qu’il était en cours pour le garçon. Une réduction sanglante suivie de l’ostéo-suture au fil non résorbable (Figure 5, Figure 6, Figure 7) ou du brochage a connue des suites simples avec une ablation de la broche à la 8^ème^ semaine. L’épaule était libre et indolore. Il a été relevé une cicatrice hypertrophique à tendance chéloïdienne. Au recul moyen de 24 mois (extrêmes 17 et 26 mois), une évaluation clinique selon le score d’ESKOLA a donné un score moyen de 10.2/ 12 points (extrêmes 9 et 11).

## Discussion

### Epidémiologie

La clavicule est le premier os long à s’ossifier dès la 5^ème^ semaine de vie intra-utérine. Elle est aussi, par son épiphyse médiale, la dernière à apparaître, à s’ossifier et à fusionner de tout le squelette. Cette fusion s’effectue entre 23 et 25 ans [2]. Le cartilage de conjugaison constitue le maillon le plus faible de ce site articulaire [2, 7]. Cette fragilité explique la survenue des fractures-décollements épiphysaires chez les enfants et adolescents contrairement au squelette mature de l’adulte qui présenterait, en lieu et place, une vraie luxation sterno-claviculaire [8]. La fracture-séparation de l’épiphyse médiale de la clavicule ne serait décrite dans la littérature anglaise que depuis 1967 par Denham [9]. Elle est régulièrement confondue à l’étape clinique et radiologique conventionnelle avec la luxation sterno-claviculaire et la fracture du 1/ 3 médial de la clavicule. Elle est fréquemment décrite sous la forme d’un fait Clinique [1, 7, 8, 10–13]. Elle a représenté 0,44% de l’ensemble des 225 fractures-décollements épiphysaires suivies dans notre service de mars 2003 à mars 2009 [14]. Les treize (13) cas de Lafosse [15] tirés de sa série de 30 disjonctions sterno-claviculaires constituent, à notre connaissance, la plus importante série publiée ces 10 dernières années (Tableau 2). La prédominance masculine est la règle: le rapport garçons/filles est pour nous de 5/1, 11/2 pour Laffosse [15] et 6/1 pour Tennent [16]. Gobet [3] reste le seul auteur à avoir rapporté autant de filles que de garçons (3/3). Il s’agit d’une lésion notée le plus souvent chez l’adolescent et l’enfant [2, 15, 16]. Elle peut toucher le nouveau-né à l’occasion d’un traumatisme obstétrical mais épargne le nourrisson.

**Tableau 2:** Revue de la littérature

Auteurs	Ville / Année	Cas	Période	Sexe		Age			Accident		Agr.	Tr. O
				M	F	Nné	Enft	Ado	AcVC	ACR		
Goldfarb	Missouri, 2001	6	4 ans	6	0	0	1	5	5	1	0	
Gobet	Zurich, 2004	6	4 ans	3	3	0	5	1	5	1	0	
Tennent	London,2012	7	7 ans	6	1	0	0	7	6	0	1	
Laffosse	Toulouse,2010	13	13 ans	11	2	0	0	13	13			
Sy	Dakar, 2012	6	2 ans	5	1	1	1	4	3	1	1	1

AcVC : Accident de la vie courante ; AC : Accident de la circulation;

Agr : Agression ; Enft: Enfant ; Ado : Adolescent ; Tr O : Traumatisme obstétrical

### Circonstances et mécanisme lésionnels

Cette prédominance dans l’adolescence serait, en partie, liée à l’hyperactivité et à la pratique de jeux ou de sports parfois dangereux propres à cette tranche d’âge: rugby, lutte, planche à roulettes… d’où une nette prépondérance des accidents de la vie courante [2, 15–17]. Le mécanisme lésionnel serait double ressemblant à celui de la luxation. Il peut s’agir d’un impact direct un impact direct sur la clavicule repoussée en arrière du sternum dans les forme postérieure. A l’opposé, il peut s’agir d’un mécanisme indirect: une chute sur le moignon de l’épaule déjeté soit en avant (antépulsion avec une réduction de la distance acromio-sternale) dans la forme postérieure ou en arrière (rétropulsion avec une réduction de la distance acromio-sternale) dans la forme antérieure.

### Diagnostic

Elle est suspectée dans sa forme isolée cliniquement devant une dépression post-traumatique douloureuse à l’endroit de l’articulation sterno-claviculaire (forme postérieure ou rétro-sternale) ou une tuméfaction saillante (forme antérieure). Le diagnostic lésionnel précisest difficile avec la radiographie conventionnelle car il existe une superposition des structures anatomiques [15]. Le recours à la tomodensitométrie est indispensable pour une confirmation du diagnostic, de la nature de la lésion et d’éventuelles complications [7, 8, 17]. Sferoupolos [18] et Benitez [19] signalent le rôle respectif de l’échographie et de l’IRM dans le diagnostic de ces lésions.

### Formes cliniques

Les formes cliniques restent dominées par les particularités de la forme du nouveau-né, les formes anatomo-cliniques surtout topographiques postérieures, les formes compliquées et celles associées à d’autres lésions ipsi-latérales de la ceinture scapulaire.

### Forme néo-natale

Elle fait suite à un accouchement presque toujours dystocique par voie basse tel aussi dans le cas de Beluffi [20]. Notre forme était particulière par la présence d’un callus périosté exubérant trompeur en situation médio-claviculaire issu du « déchaussement périosté ». Il devait être distingué d’un cal hyperthrophique d’une fracture du 1/3 moyen.

### Forme rétro-sternale

Le déplacement du moignon claviculaire peut se faire en avant avec un risque d’embrocher la peau ou en arrière derrière le sternum avec un risque de compression des éléments vasculo-nerveux et aéro-digestifs du médiastin supérieur. Cette forme dite rétro-sternale est la plus fréquente autant dans les courtes séries [2, 15, 19] que dans les cas uniques rapportés [7, 8, 10, 11, 13]. Sur un total de 44 cas réunis entre 1989 et 2013 à travers la littérature, on note 84% (n=37) de formes postérieures contre 16% (n=7) de formes antérieures (Tableau 3). Elles restent très graves (compression vasculaire veineuse plus rarement artérielle) souvent symptomatiques (Odynophagie, raucité de la voix..) et menacent le pronostic vital.

**Tableau 3:** Formes anatomo-cliniques / revue littérature

Auteurs	Ville / Année	Cas	Déplacement	
			Antérieur	Postérieur
Goldfarb	Missouri, 2001	6	0	6
Gobet	Zurich, 2004	6	3	3
Tennent	London,2012	7	0	7
Laffosse	Toulouse,2010	13	0	13
Sy	Dakar, 2013	6	3	3
Lewonowski	Los Angeles,1992	1	0	1
Carmichael	Texas,2006	1	0	1
Leighton	Paris,1989	1	0	1
Beecroft	Chigago, 2007	1	0	1
Mekkaoui	Saint Denis,2011	1	1	0
Lehnert	Frankfort, 2005	1	0	1
Total		44	7	37
Pourcentage			16	84

### Décollement médial associé à une fracture ipsilatérale de la clavicule (Tableau 4)

Le décollement de l’épiphyse médiale peut survenir de façon concomitante à une luxation acromio-claviculaire [21] ou à une fracture de la clavicule ipsilatérale. Cette dernière association est souvent retrouvée chez l’adolescent victime d’une chute sur la ceinture scapulaire à gauche [12, 22–26]. Le déplacement du fragment libre se fait en pivotant en arrière d’au moins 120° à partir du foyer de fracture situé à l’union du 1/3 médial et du 1/3 moyen ou en médio-claviculaire.

**Tableau 4:** Décollement epiphyse médiale associée à une fracture ipsi-latérale de la clavicule/ revue litérature

Ville / Année	Cas	Sexe	Age	Accident	Clavicule	DE + Fx Cl.	Complications
**Montréal, 1984**	1	G	15	Sportif(Hockey)	Gauche	Salter I +1/3 Mu 1/3M	FF comprimant VSC et plèvre
**Pennsylvania,2006**	2	G	17	Sportif(SnowBoard)	Gauche	Salter I +1/3 Mu 1/3M	Déplacement vertical
			15	Chute/Rixe	Droite	Salter I +1/3 Mu 1/3M	Déplacement vertical
**Lille, 2007**	1	G	16	Chute/ Moto	Gauche	Salter I +1/3 Mu 1/3M	Déplacement Horizontal 150°
**Bologna, 2008**	1	G	14	Chute/football	Gauche	Salter I +1/3 Mu 1/3M	Déplacement Horizontal 150°
	1	F	7	Chute/ cheval	Droite	Salter I +1/3 M	Angulation
**Dakar, 2013**	1	G	19	Chute/ Rixe	Gauche	Salter I +1/3 Mu 1/3M	Déplacement Horizontal 170° Compression VSC

1/3 Mu 1/3M : Tiers moyen union tiers médial

VSC : Veine sous-clavière FF : Fracture fermée

### Complications

La complicationla plus fréquente de la fracture-décollement de l’épiphyse claviculaire médiale est constituée par les lésions de la veine sous-clavière [27]: compression, obstruction et rupture vasculaire. Nous en avons noté 2 cas de compression asymptomatique. Les autres complications post-traumatiques peuvent être la dyspnée par compression trachéale [15]ainsi que la dysphagie par compression de l’œsophage [3, 15]. La complication post-opératoire redoutée reste la migration des broches vers le médiastin, le cœur [28, 29].

### Considérations thérapeutiques

La réduction orthopédique suivie d’une contention par bandage reste réservée aux seules formes antérieures et néo-natales. Une tendance nette se dégage vers une indication chirurgicale quasi constante devant la forme rétro-sternale et la forme associée à une fracture ipsilatérale de la clavicule. La réduction est sanglante par abord direct. Les modalités de l’ostéosynthèse sont différentes suivant le caractère isolée ou associée de la lésion. L’ostéosynthèse de la fracture-décollement isolée peut être assurée par des points trans-osseux ou par des broches. La préférence est à l’ostéosuture pour beaucoup d’auteurs [16, 17, 30–31]. Elle présente de nombreux avantages liés à la relative facilité, la sécurité (pas de risque de migration), le coût et dispense le malade de la ré-intervention. Plusieurs modalités techniques sont proposées: 2 à 4 points de sutures trans-osseux de part et d’autre du foyer au fil d’acier pour Goldfarb [17] et Thomas [31] ou au fil non métallique pour Hofwegen [30]; 4 à 8 points entre d’une part l’os et d’autre part le tissu fibro-périosté au fil non métallique pour Tennent [16] et pour Nous. L’ostéosynthèse de la fracture-décollement associée à une fracture de la clavicule est réalisée aussi suivant différents moyens: La réduction-reposition du fragment avec: une ostéosuture transosseuse assurée par des points trans-osseux pour Lemire [26]; Un haubannage pour Falcone [23] ; Une ostéosynthèse par plaque vissée pour Segal [25] et Lampasi [24]. Les plaques vissées constituent une bonne butée contre la récidive, n’exposent pas au risque de migration dangereuse dans le médiastin, mais présentent l’inconvénient de la ré-intervention pour ablation du matériel. La résection du fragment suivie d’une suture ostéo-périostée stabilisant le moignon claviculaire avec un tube périosté et une préservation du ligament costo-claviculaire telle proposée par Rockwood [32] et Panzica [33] a été réalisé chez un de nos patients avec un bon résultat clinique.

## Conclusion

La fracture-décollement de l’extrémité médiale de la clavicule mime au plan clinico-radiologique la luxation sterno-claviculaire. Elle reste grave dans sa forme rétro-sternale et associée à une fracture ipsi-latérale de la clavicule. Ces formes peuvent engager le pronostic vital par le risque de compression viscérale et vasculaire. La TDM et l’IRM restent irremplaçables pour un diagnostic précis. La suture ostéo-périostée au fil non résorbable constitue une technique chirurgicale sure, accessible et reproductiblequi procure de bons résultats.

### Etat des connaissances actuelles sur le sujet

- La fracture-décollement de la clavicule médiale est une lésion rare souvent méconnue ou confondue à la luxation sterno-claviculaire;
- Le risque de compression médiastinale dans les formes postérieures en fait toute la gravité et traitement dans ses modalités pratiques reste controversé.

### Contribution de notre étude à la connaissance

- Précise la place des formes néonatales dans l’épidémiologie de cette lésion;
- La suture ostéopériostée constitue une modalité de traitement sure et alternative à la fixation métallique sans les inconvénients de cette dernière;
- Le scanner et l’IRM sont irremplaçables pour un diagnostic précis.
